# Bullous pemphigoid‐like skin rash associated with Rivaroxaban use in a very elderly patient with multimorbidity and chronic kidney disease: A case report

**DOI:** 10.1002/ccr3.2757

**Published:** 2020-02-26

**Authors:** Shakil Ahmed Chohan, Diraviyam Balasubramanian, Samuel Ee

**Affiliations:** ^1^ Changi General Hospital Singapore Singapore

**Keywords:** bullous pemphigoid, chronic kidney disease, rivaroxaban, skin

## Abstract

Direct oral anticoagulants (DOACs) are increasingly being used in the elderly population in view of its ease of use, efficacy, and favorable side‐effect profile compared with the vitamin K antagonists. However, there is a need for increase awareness of well‐characterized cases, albeit a small number, of bullous pemphigoid‐like skin reactions associated with rivaroxaban.

## Graphical abstract

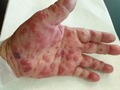

## 1 INTRODUCTION

Direct oral anticoagulants (DOACs) are commonly used in the treatment of atrial fibrillation and venous thromboembolism, among other indications. They offer a potential alternative to older agents such as low‐molecular weight heparin and vitamin K antagonists. DOACs are generally well tolerated with good side‐effect profiles.^1^ rivaroxaban is an example of a DOAC, and apart from bleeding, less common side effects include hepatobiliary toxicity, hair loss, skin reactions, and hypersensitivity reactions including anaphylaxis.^2^ There have been a number of reports of rash occurring with rivaroxaban use.^3^, ^4^, ^5^, ^6^ A bullous‐like skin rash has been described in some of these studies,^7^ which may be mistaken for the condition of bullous pemphigoid. Manufacturers warn of the following dermatologic adverse reactions in the package insert of wound secretions (3%), pruritus (2%), and skin blister (1%). Cutaneous drug eruptions are among the most common adverse reactions and may represent a challenging diagnostic problem. Although the temporal link between initiation of drug therapy and the onset of drug rash is critical for diagnosis, drug reactions may also occur during chronic drug administration, evolve over time, and represent a diagnostic challenge in the very elderly population with multimorbidity.

## 2 CASE DESCRIPTION

We describe a case of an 86‐year‐old Chinese male, with past medical history of ischemic heart disease, heart failure, type 2 diabetes mellitus, hyperlipidemia, cerebrovascular disease with secondary expressive dysphasia, chronic kidney disease (CKD), chronic obstructive pulmonary disease (COPD), and osteoarthritis of the knees. He also has a history of behavioral and psychological disorder of dementia and recurrent falls from a lack of safety awareness. His weight taken on 19 June 2019 was 62.6 kg with a height of 1.60 cm, giving a body mass index of 24.4 kg/m^2^. The patient was on the following oral medications (Box 1) long term prior to the addition of rivaroxaban 10 mg OM for chronic atrial flutter and previous ischemic stroke.

Box 1 **Patients regular medication**

**Table:** 

Drug	Dose and frequency
Amiodarone	200 mg OM
Digoxin	62.5 µg OM
Furosemide	20 mg OM
Spironolactone	12.5 mg OM
Gliclazide MR	60 mg OM
Aspirin	100 mg OM (stopped on initiation of rivaroxaban)
Omeprazole	20 mg OM
Cholecalciferol	1000 units OM
Salbutamol inhaler (100 µg)	2 puffs QDS
Ipratropium inhaler (20 µg)	2 puffs QDS
Salbutamol Neb Solution (0.5%)	1 mL BD PRN
Ipratropium bromide (0.025%)	1 mL BD PRN

He had an episode of severe infective exacerbation of COPD as a result of postviral pneumonia, which was complicated by septic shock, multiorgan failure, non‐ST elevation myocardial infarction, and new anemia in December 2018, for which he was admitted to the intensive care unit for inotrope support and mechanical ventilation for respiratory failure. The liver and respiratory function recovered, but the renal function did not and led to new chronic kidney disease,^8^ with an estimated glomerular filtration rate of 31 mL/min by Cockcroft‐Gault equations on 19 June 2019. He developed new atrial flutter which persisted but was not started on oral anticoagulation immediately due to concerns with regards the new multiorgan failure and unexplained new anemia in the intensive care unit. He was eventually started on rivaroxaban on 17 April 2019 after physical recovery to his premorbid state with three months of rehabilitation and medical review at the local geriatric day hospital and stabilization of his hemoglobin and renal function.

**Figure 1:**
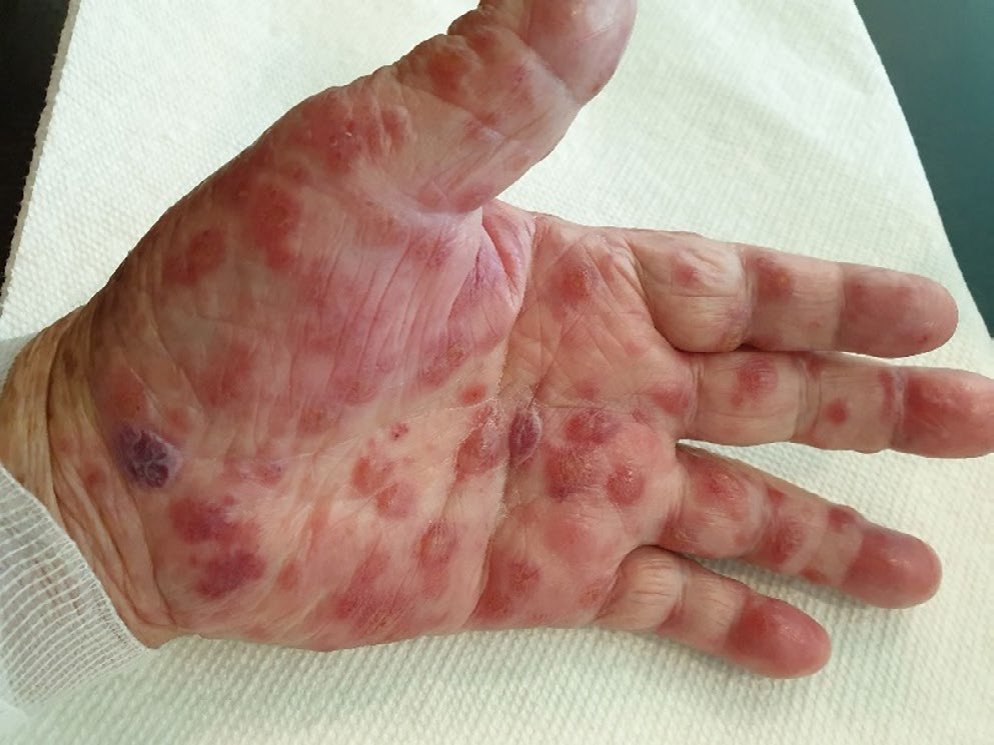
Left hand (14 July 2019)

**Figure 2:**
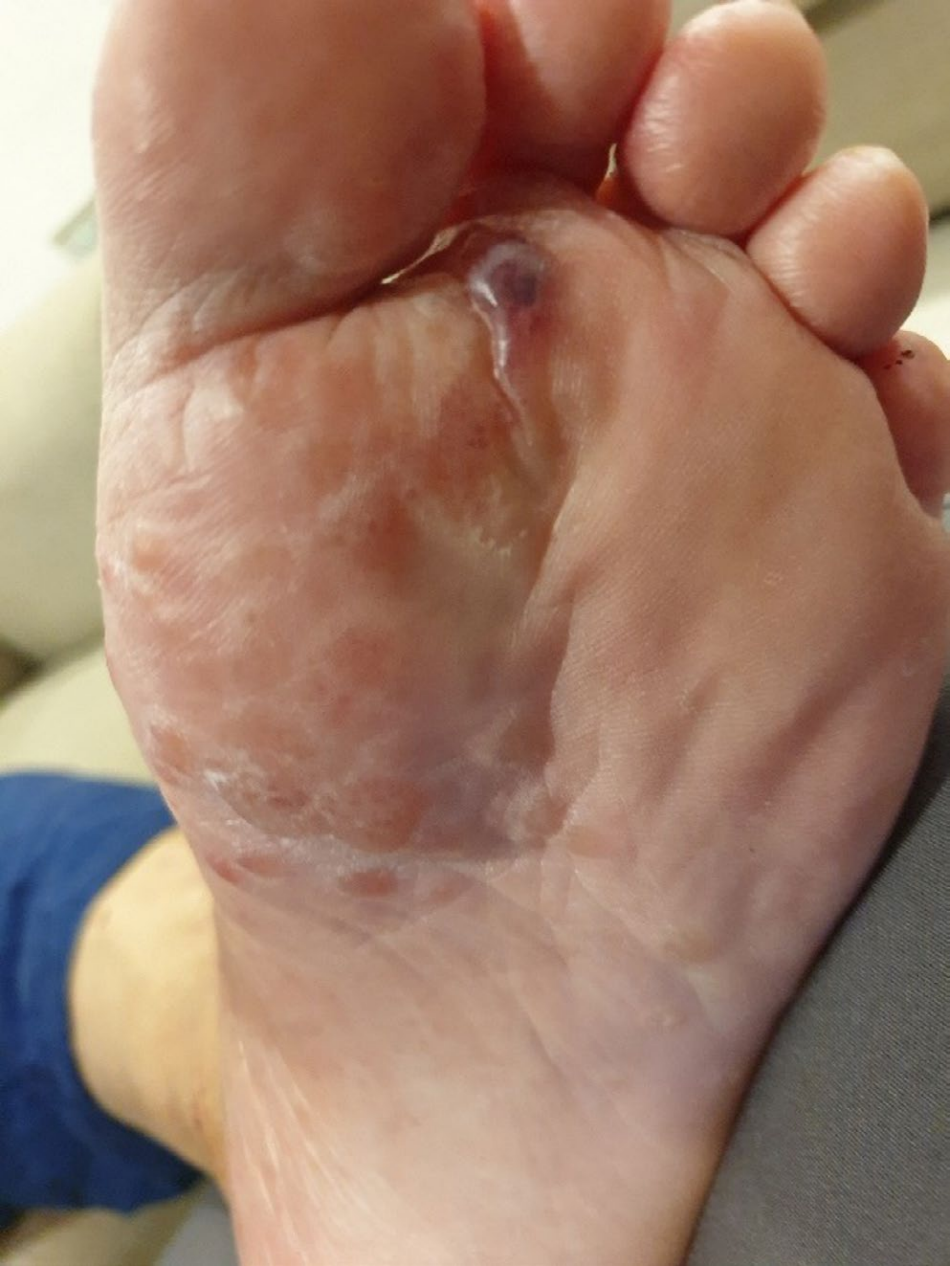
Left foot (15 July 2019)

He presented to Dermatology Department 12 weeks later on 11 July 2019 with complaints of new papular rash over the palmar aspects of both hands with small vesicles over 1‐week duration (Figure 1). Possible differentials of acral pompholyx, bullous pemphigoid, and scabies were considered. Blood results on 16th July as per in Box 2. His eGFR by Cockcroft‐Gault formula was 32 mL/min. He had serum tested for indirect immunofluorescence and was empirically treated with permethrin, and topical clobetasol for the palms and 0.1% betamethasone for the scattered pruritic macular rash on the trunk. Subsequently, the vesicles evolved into blisters and involved the soles of both feet despite the topical steroids (Figure 2). There was no involvement of the mouth or other mucosal areas.

Box 2 **Blood results 16th July 2019**

**Table:** 

Blood test	Values	Reference range
Hemoglobin	11.0	13.0‐17.0 g/dL
White cell count	14.7	4.0‐10.0 × 10 3/μL
Neutrophil absolute	9.7	2.0‐7.5 × 10 3/μL
Lymphocyte absolute	2.2	1.0‐3.0 × 10 3/μL
Monocyte absolute	0.9	0.2‐0.8 × 10 3/μL
Eosinophil absolute	1.9	0.0‐0.4 × 10 3/μL
Platelet count	242	150‐450 × 10 3/μL
Serum urea	11.0	2.8‐7.7 mmol/L
Serum creatinine	128	65‐125 umol/L
Serum bilirubin	6.4	5.0‐30.0 umol/L
Alanine transaminase	18	10‐55 U/L
AST	14	20‐36 U/L
CRP	84.1	<3.0 mg/L

**Figure 3:**
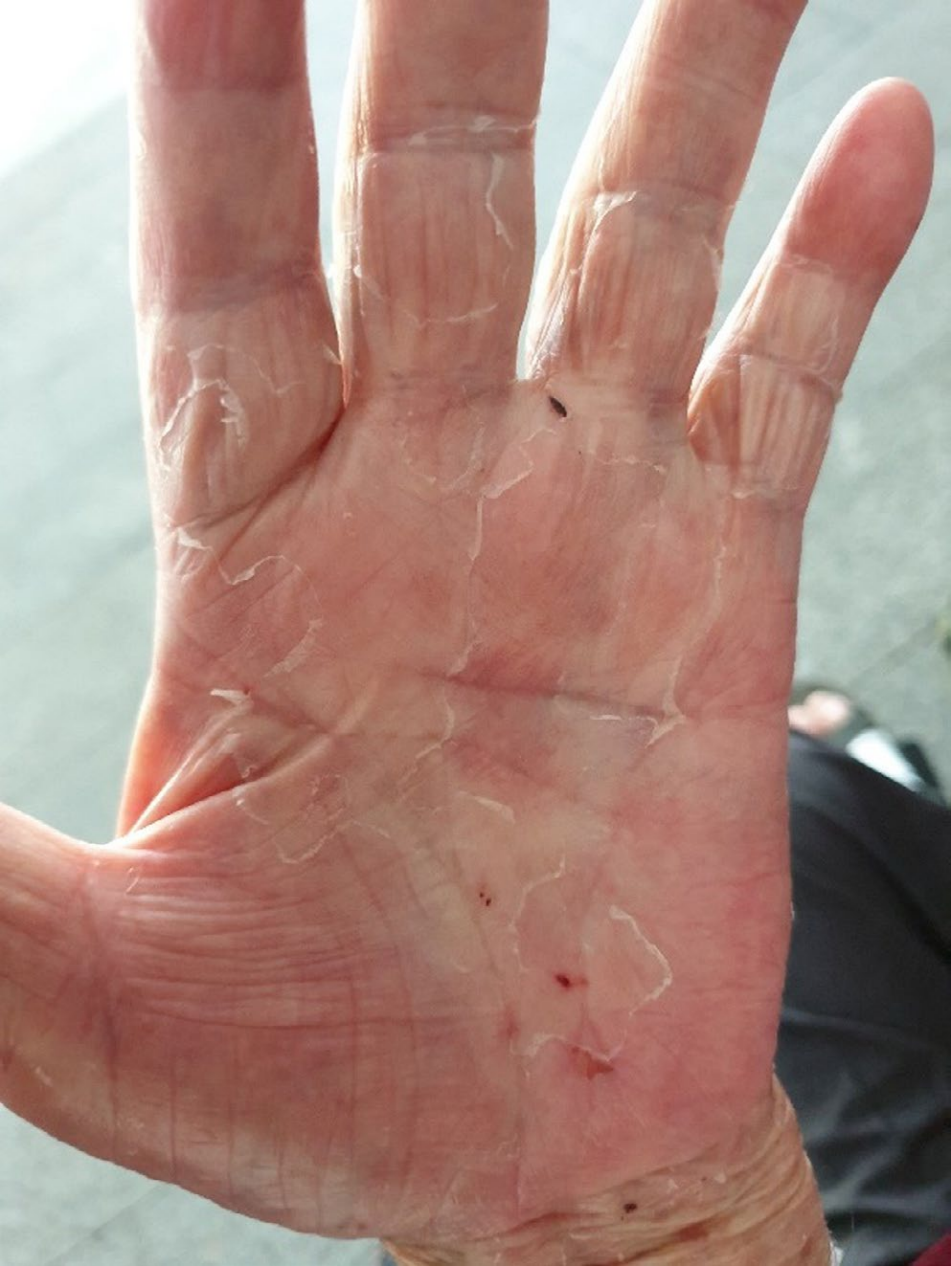
Left hand (27 July 2019)

**Figure 4:**
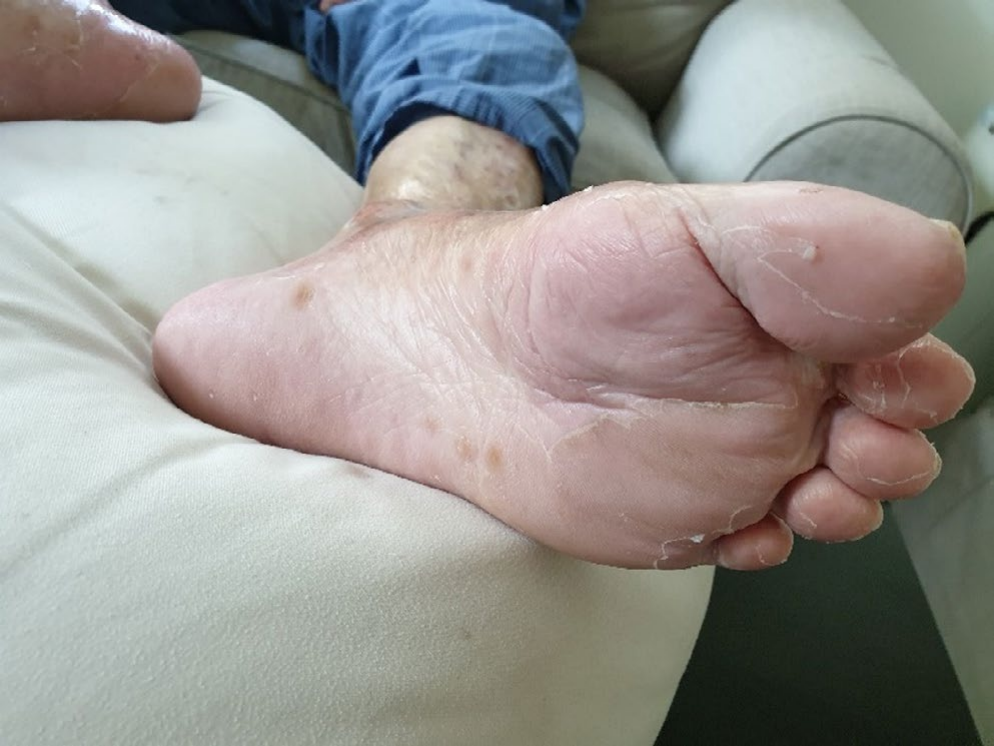
Left foot (2 August 2019)

After reviewing the published case reports on skin reactions associated with rivaroxaban,^3^, ^4^, ^5^, ^6^, ^7^ a decision was taken to suspend rivaroxaban on 17 July 2019. Subsequently, the patient and his family did not want any further anticoagulation medication in view of the complications with rivaroxaban and his history of recurrent falls with physical injuries. This was followed by rapid improvement in both pruritic symptoms, cessation of formation of new blisters and resolution of pre‐existing blisters over the next 7 to 10 days. Indirect immunofluorescence of serum for BP 180 and BP 230 was not suggestive of bullous pemphigoid. Skin biopsy was not performed in view that lesions had shown improvement with cessation of rivaroxaban. Complete resolution of both palmar and sole blistering lesions was achieved by about 2 weeks after suspension of rivaroxaban on 27 July 2019 for the hands (Figure 3) and 2 August 2019 for the feet (Figure 4). Rivaroxaban has remained suspended, and patient remains well off all topical steroids, with no further recurrence of either pruritic macular rashes or blistering lesions as of 25 September 2019, almost 2 months after complete resolution of the skin lesions.

On retrospective enquiry of the patient's live‐in carer, the patient was noted to have generalized pruritus about a week after rivaroxaban was started, with a gradually increasing intensity over time, with a macular papular rash evolving over time. As nonspecific pruritus is not uncommon in the very old patients, and the patient has expressive dysphasia from his previous stroke, the significance of this early increased pruritus was not noticed and was managed with topical moisturizers only. Topical 0.1% betamethasone was started by the dermatologist on 11 July 2019.

## 3 DISCUSSION

A dermatological reaction is recognized complication of rivaroxaban and is listed in the summary of product characteristics. We performed a search using the keywords “rivaroxaban” and “rash” in the Food and Drug Administration (FDA) Adverse Drug Event Reporting System (FAERS) and noted that there are over 1000 reported cases. When we repeated the search using the keywords “rivaroxaban” and “pemphigoid,” we noted that are more than 25 cases reported in the system.^9^

These reactions can manifest along a continuum of a pruritic skin rash to bullous pemphigoid‐like lesions. The skin blisters reported in this case with the use of rivaroxaban would be difficult to differentiate from bullous pemphigoid, with both conditions even having similar findings on skin biopsy. The negative serum immunofluorescence tests for bullous pemphigoid and the improvement of symptoms with withdrawal of rivaroxaban make drug‐induced bullous pemphigoid‐like skin eruption a likely diagnosis in our patients’ case. The Naranjo score^10^ was 7 (Box 3), indicating a probable probability that the drug reaction was related to the use of rivaroxaban.

Box 3 **Naranjo Algorithm—ADR Probability scale**

**Table:** 

Question	Yes	No	Do not know	Score
1. Are there previous conclusive reports on this reaction?	+1			1
2. Did the adverse event appear after the suspected drug was administered?	+2			2
3. Did the adverse event improve when the drug was discontinued or a specific antagonist was administered?	+1			1
4. Did the adverse event reappear when the drug was readministered?			0	0
5. Are there alternative causes that could on their own have caused the reaction?		+ 2		2
6. Did the reaction reappear when a placebo was given?			0	0
7. Was the drug detected in blood or other fluids in concentrations known to be toxic?			0	0
8. Was the reaction more severe when the dose was increased or less severe when the dose was decreased?			0	0
9. Did the patient have a similar reaction to the same or similar drugs in any previous exposure?			0	0
10. Was the adverse event confirmed by any objective evidence?	+1			1
	Total score: 7

Hypersensitivity reactions can range from type I to type IV,^11^ and drug reactions can be further classified into Type A, or predictable medication side effects and Type B, or unpredictable side effects, which often include immunogenic and allergic reactions. Drug hypersensitivity reactions can be either immediate (whereby they can present with symptoms of angioedema, urticarial, bronchospasm, or anaphylaxis), or delayed, in which presentation can be varied. Skin manifestations (including bullous eruptions) are considered to be delayed drug reactions.^12^

It is possible that our patient developed a Type IV hypersensitivity reaction to rivaroxaban. The exact pathophysiology for drug‐induced severe cutaneous reactions is not completely understood. It is possible that drugs (including rivaroxaban) form haptens by directly binding with serum or intracellular proteins which are then presented on the surface of antigen‐presenting cells bound to human leukocyte antigen (HLA). This is then recognized and bound by the T‐cell receptors (TCR), leading to T‐cell activation. Two other models proposed involve the “pharmacological interaction” between drug and the HLA molecule without the formation of covalent bonds, and the altered peptide model where the noncovalent binding of the drug molecule to the HLA molecule exposes hidden epitopes to the T cells. These two models are thought to be responsible for the severe cutaneous reactions involving carbamazepine and Abacavir, respectively.^13^

In bullous pemphigoid, the pathophysiology is due to an autoimmune response to the anchoring hemidesmosomal proteins within the dermal‐epidermal junction. They are designated BP180 and BP 230, with BP180 being the main antigen.^14^ Binding of IgG autoantibodies with these cutaneous basement membrane proteins leads to formation of antibody‐antigen complexes which then triggers complement activation, mast cell degranulation, and accumulation of neutrophils and eosinophils. Subsequent release of proteases by granulocytes leads to dermal‐epidermal separation. Mast cell degranulation recruits neutrophils to the site, and disease severity is subsequently determined by the number of infiltrating neutrophils to the site. Eosinophils are thought to contribute to the initiation and progression of the inflammatory process.^15^

It is thought that a variety of factors leads to a loss of immune tolerance for the hemidesmosomal proteins. These include predisposing HLA allotype, UV radiation, trauma, and drugs. There are two types of drug‐related BP, namely drug‐induced BP and drug‐triggered BP. In the former, the removal of the offending drug leads to the resolution of BP but not in the later. The offending drugs can be topical or systemic in nature.^16^ There is also a known association between BP and neurological conditions such as stroke, dementia, and Parkinson's disease,^15^, ^16^ although the pathophysiology for this relationship is still unknown.

Plasma rivaroxaban levels are raised in CKD. In patients with a clearance of <30 mL/min, the plasma levels are expected to rise by up to 1.6‐fold.^17^ This would increase the risk of bleeding as well as other rivaroxaban related adverse drug reactions. In our patient, his estimated GFR by CG is 32.18 mL/min on 19 Jun 2019 and would have contributed to the increased risk of rivaroxaban related skin reaction.

Rivaroxaban is metabolized by the CYP3A4 and P‐glycoprotein pathways. Strong inhibition of both of these pathways concurrently by azole antimycotics or HIV protease inhibitors has demonstrated an increase of up 2.6‐fold in mean rivaroxaban area under curve (AUC) and a 1.7‐fold increase in mean rivaroxaban Cmax. Drugs strongly inhibiting only one of the two pathways, such as clarithromycin, will lead to a more modest rise in plasma rivaroxaban concentrations. Drugs moderately inhibiting both pathways such as erythromycin will also lead to a more modest rise in AUC and Cmax for rivaroxaban which may only become significant in CKD.^17^

The patient in our case report had been receiving amiodarone since the onset of atrial flutter with fast ventricular rate on 26 Dec 2018. Amiodarone is a known moderate inhibitor of both CYP3A4 and P‐glycoprotein. The drug‐drug interaction between amiodarone and rivaroxaban is expected to lead to an increase of AUC for rivaroxaban ≥twofold but ≤fivefold.^18^ In our patient, his eGFR approaches the threshold for CKD Stage 4, and this would have further increased the rise in plasma levels in an additive manner.

There are no known clinically significant drug‐drug interactions between rivaroxaban and omeprazole and between rivaroxaban and digoxin.^19^

Although not performed in our patient, patch testing and lymphocyte transformation test (LTT) are two tools which may help confirm the presence of rivaroxaban allergy. LTT uses antigen to stimulate drug‐specific memory T cells to proliferate. The sensitivity and specificity for both patch testing and LTT have been quoted to be 78% and 85% for LTT and 64% and 85% for patch testing, respectively.^20^

## 4 CONCLUSION

There is growing impetus to use the perceived safer and patient friendly, direct oral anticoagulants particularly for older and very old patients. However, this needs to be balanced by the awareness that with increasing multimorbidity, polypharmacy, and prevalence with age, there will also be an increased risk associated with drug‐drug interactions via CYP3A4 and P‐glycoprotein pathways. This is especially so with the strong inhibitors of both CYP3A4 and P‐glycoprotein, and in patients with chronic kidney disease, less strong inhibitors as well. Although there are only a few cases of cutaneous reaction associated with rivaroxaban, a high level of suspicion and an awareness of this possibility are required for early diagnosis and management as it may be completely reversible in the early stages as in our case.

## CONFLICT OF INTEREST

None declared.

## AUTHOR CONTRIBUTIONS

SAC: was the lead author involved in writing the first draft, subsequent revisions, and literature search; DB: was the second author who was involved with contributions to write up, revisions, and literature search; SE: was the third author who was involved with contributions to write up, revisions, and literature search.
